# Dibromido[(*tert*-butyl­amino)dimeth­yl(piperidin-1-ylmeth­yl)silane-κ^2^ *N*,*N*′]zinc(II)

**DOI:** 10.1107/S1600536809018364

**Published:** 2009-05-23

**Authors:** Victoria P. Colquhoun, Carsten Strohmann

**Affiliations:** a Anorganische Chemie, Technische Universität Dortmund, Otto-Hahn-Strasse 6, 44227 Dortmund, Germany

## Abstract

The title compound, [ZnBr_2_(C_12_H_28_N_2_Si)], is an example of a neutral coordination compound of a bidentate ligand to a metal centre with the Zn atom being coordinated by two Br and two N atoms, yielding a slightly distorted tetra­hedral coordination environment.

## Related literature

For the synthesis and structure of *cis*-(2-amino-1,1-dimethyl­ethylamine)dichloro­palladium(II) ethanol hemisolvate, see: Farrugia *et al.* (2001 ▶). For niobium and tantalum complexes of silylamides, see: Herrmann *et al.* (1992 ▶). For the synthesis and structure of ^*t*^Bu_2_Si=N-SiCl^*t*^Bu_2_, see: Lerner *et al.* (2005 ▶); for syntheses, structures and properties of chiral zinc halide catalysts, see: Mimoun *et al.* (1999 ▶). For the structure and reactivity of lithia­ted benzyl­silanes, see: Ott *et al.* (2008 ▶). For syntheses and structures of bis­{[diphen­yl(piperidinometh­yl)­sil­yl]meth­yl}cadmium and -magnesium, see: Strohmann & Schildbach (2002 ▶). For a highly diastereomerically enriched, silyl-substituted alkyl lithium, see: Strohmann *et al.* (2005 ▶). For the synthesis and structure of a monolithia­ted allyl­silane and its related 1,3-dilithia­ted allyl­silane, see: Strohmann *et al.* (2006 ▶). For the synthesis and structure of a lithia­ted [(benzyl­silyl)meth­yl]amine, see: Strohmann *et al.* (2002 ▶).
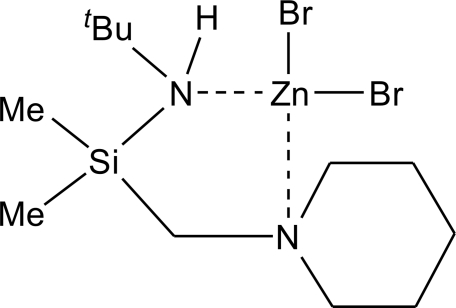


## Experimental

### 

#### Crystal data

[ZnBr_2_(C_12_H_28_N_2_Si)]
*M* *_r_* = 453.64
Monoclinic, 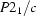

*a* = 12.0284 (4) Å
*b* = 10.6505 (3) Å
*c* = 14.5633 (5) Å
β = 109.752 (4)°
*V* = 1755.91 (10) Å^3^
*Z* = 4
Mo *K*α radiation
μ = 6.01 mm^−1^
*T* = 123 K
0.40 × 0.20 × 0.20 mm

#### Data collection

Oxford Diffraction Xcalibur S diffractometer
Absorption correction: multi-scan (*CrysAlis RED*; Oxford Diffraction, 2006 ▶) *T*_min_ = 0.698, *T*_max_ = 1.000 (expected range = 0.210–0.301)
17826 measured reflections
3440 independent reflections
2673 reflections with *I* > 2σ(*I*)
*R*_int_ = 0.034

#### Refinement

*R*[*F* ^2^ > 2σ(*F* ^2^)] = 0.020
*wR*(*F* ^2^) = 0.035
*S* = 1.04
3440 reflections
172 parameters
H atoms treated by a mixture of independent and constrained refinement
Δρ_max_ = 0.56 e Å^−3^
Δρ_min_ = −0.43 e Å^−3^

### 

Data collection: *CrysAlis CCD* (Oxford Diffraction, 2006 ▶); cell refinement: *CrysAlis RED* (Oxford Diffraction, 2006 ▶); data reduction: *CrysAlis RED*; program(s) used to solve structure: *SHELXS97* (Sheldrick, 2008 ▶); program(s) used to refine structure: *SHELXL97* (Sheldrick, 2008 ▶); molecular graphics: *ORTEP-3* (Farrugia, 1997 ▶); software used to prepare material for publication: *SHELXL97*.

## Supplementary Material

Crystal structure: contains datablocks global, I. DOI: 10.1107/S1600536809018364/fi2078sup1.cif

Structure factors: contains datablocks I. DOI: 10.1107/S1600536809018364/fi2078Isup2.hkl

Additional supplementary materials:  crystallographic information; 3D view; checkCIF report

## Figures and Tables

**Fig. 1.**
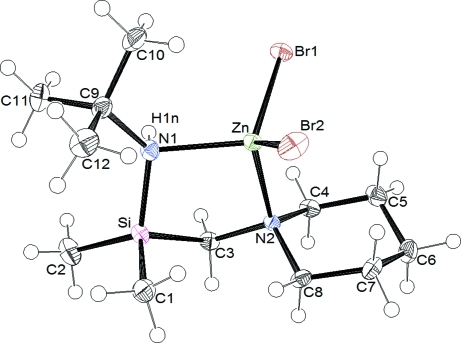
Plot of the asymmetric unit of the title compound with displacement ellipsoids drawn at the 50% probability level.

## supplementary crystallographic information

### Comment

The title compound, the adduct of the silazane ligand and zinc bromide crystallized from acetonitrile in the monoclinic crystal system, space group *P*2_1_/*c*. The H atom H1n was refined freely. It is connected to N1 with a bond length of 0.853 (21) Å which is in the expected range for N—H bonds. Additionally, H1N is forming a weak intermolecular hydrogen bond to Br1^i^ (i: –*x*, –*y* + 1, –*z* + 1). The H1···Br1^i^ distance (2.828 (22) Å) and the N1—H1N—Br1^i^ angle (166.1 (20) Å) are in the typical ranges of such hydrogen bonds (Farrugia *et al.*, 2001). With a value of 1.791 (2) Å, the Si—N bond length is in the upper range of other known systems and is very close to the sum of the covalent radii of silicon and nitrogen (1.86 Å) (Lerner *et al.*, 2005; Herrmann *et al.*, 1992). The bond lengths of 2.128 (2) Å for N1—Zn and 2.110 (2) Å for N2—Zn are similar to other reported dative zinc-nitrogen bonds (Mimoun *et al.*, 1999). The structure of the title compound is a neutral coordination compound of a bidentate ligand and zinc(II) bromide forming a five-membered ring with a typical envelope conformation similar to other known metalla heterocycles (Strohmann *et al.* 2002, 2005, 2006; Strohmann & Schildbach 2002; Ott *et al.* 2008). The tip of the envelope is formed by the Si atom with a distance of 0.8312 (7) Å to a least-squares plane through Zn, N1, N2, C3 and Si. The title compound may be regarded as a comparative model structure for a deprotonation transition state as the silazane ligand can also be deprotonated by more reactive organozinc reagents. Thereby new metal silazane compounds are formed which themselves are interesting as deprotonation or alkylation reagents in organic synthesis.

### Experimental

To 0.38 g (1.7 mmol) dry zinc(II) bromide dissolved in 10 ml dry acetonitrile, 0.38 g (1.7 mmol) *N*-*tert*-butyl-1,1-dimethyl-1-(piperidin-1-ylmethyl)silanamine were added and stored at room temperature. After 24 h a colourless crystalline solid of the title compound suitable for single-crystal *x*-ray studies had formed.

### Refinement

The H atoms were refined in their ideal geometric positions using the riding model approximation with *U*_iso_(H) = 1.5*U*_eq_(C) for methyl H atoms and of *U*_iso_(H) = 1.2*U*_eq_(C) for all other H atoms except atom H1n (bonded to N1) which was refined freely.

### Crystal data

**Table:**

[ZnBr_2_(C_12_H_28_N_2_Si)]	*F*(000) = 912
*M**_r_* = 453.64	*D*_x_ = 1.716 Mg m^−^^3^
Monoclinic, *P*2_1_/*c*	Mo *K*α radiation, λ = 0.71073 Å
Hall symbol: -P 2ybc	Cell parameters from 9282 reflections
*a* = 12.0284 (4) Å	θ = 2.4–29.1°
*b* = 10.6505 (3) Å	µ = 6.01 mm^−^^1^
*c* = 14.5633 (5) Å	*T* = 123 K
β = 109.752 (4)°	Block, colourless
*V* = 1755.91 (10) Å^3^	0.40 × 0.20 × 0.20 mm
*Z* = 4	

### Data collection

**Table:**

Oxford Diffraction Xcalibur S diffractometer	2673 reflections with *I* > 2σ(*I*)
Radiation source: Enhance (Mo) X-ray Source	*R*_int_ = 0.034
graphite	θ_max_ = 26.0°, θ_min_ = 2.4°
ω scans	*h* = −14→14
Absorption correction: multi-scan (*CrysAlis RED*; Oxford Diffraction, 2006)	*k* = −13→13
*T*_min_ = 0.698, *T*_max_ = 1.000	*l* = −17→17
17826 measured reflections	1 standard reflections every 50 reflections
3440 independent reflections	intensity decay: none

### Refinement

**Table:**

Refinement on *F*^2^	Primary atom site location: structure-invariant direct methods
Least-squares matrix: full	Secondary atom site location: difference Fourier map
*R*[*F*^2^ > 2σ(*F*^2^)] = 0.020	Hydrogen site location: inferred from neighbouring sites
*wR*(*F*^2^) = 0.035	H atoms treated by a mixture of independent and constrained refinement
*S* = 1.04	*w* = 1/[σ^2^(*F*_o_^2^) + (0.012*P*)^2^] where *P* = (*F*_o_^2^ + 2*F*_c_^2^)/3
3440 reflections	(Δ/σ)_max_ = 0.002
172 parameters	Δρ_max_ = 0.56 e Å^−^^3^
0 restraints	Δρ_min_ = −0.43 e Å^−^^3^

### Special details

**Table:**

Experimental. CrysAlis RED, Oxford Diffraction Ltd. Empirical absorption correction using spherical harmonics, implemented in SCALE3 ABSPACK scaling algorithm.
Geometry. All e.s.d.'s (except the e.s.d. in the dihedral angle between two l.s. planes) are estimated using the full covariance matrix. The cell e.s.d.'s are taken into account individually in the estimation of e.s.d.'s in distances, angles and torsion angles; correlations between e.s.d.'s in cell parameters are only used when they are defined by crystal symmetry. An approximate (isotropic) treatment of cell e.s.d.'s is used for estimating e.s.d.'s involving l.s. planes.
Refinement. Refinement of *F*^2^ against ALL reflections. The weighted *R*-factor *wR* and goodness of fit *S* are based on *F*^2^, conventional *R*-factors *R* are based on *F*, with *F* set to zero for negative *F*^2^. The threshold expression of *F*^2^ > σ(*F*^2^) is used only for calculating *R*-factors(gt) *etc*. and is not relevant to the choice of reflections for refinement. *R*-factors based on *F*^2^ are statistically about twice as large as those based on *F*, and *R*- factors based on ALL data will be even larger.

### Fractional atomic coordinates and isotropic or equivalent isotropic displacement parameters (Å^2^)

**Table:**

	*x*	*y*	*z*	*U*_iso_*/*U*_eq_	
Br1	0.034333 (19)	0.34304 (2)	0.425367 (16)	0.02008 (7)	
Br2	0.36966 (2)	0.21717 (2)	0.546307 (18)	0.02735 (7)	
C1	0.43775 (18)	0.4264 (2)	0.80678 (16)	0.0251 (6)	
H1A	0.4537	0.3636	0.7637	0.038*	
H1B	0.4397	0.3863	0.8679	0.038*	
H1C	0.4980	0.4924	0.8209	0.038*	
C2	0.2739 (2)	0.6418 (2)	0.80978 (17)	0.0296 (6)	
H2A	0.3418	0.6969	0.8175	0.044*	
H2B	0.2703	0.6207	0.8742	0.044*	
H2C	0.2011	0.6850	0.7714	0.044*	
C3	0.16458 (18)	0.3884 (2)	0.74214 (15)	0.0168 (5)	
H3A	0.1720	0.3654	0.8098	0.020*	
H3B	0.0894	0.4349	0.7138	0.020*	
C4	0.03415 (18)	0.2212 (2)	0.65665 (15)	0.0178 (5)	
H4A	−0.0202	0.2816	0.6117	0.021*	
H4B	0.0116	0.2148	0.7159	0.021*	
C5	0.0203 (2)	0.0941 (2)	0.60787 (16)	0.0234 (6)	
H5A	0.0380	0.1013	0.5465	0.028*	
H5B	−0.0625	0.0656	0.5912	0.028*	
C6	0.10247 (19)	−0.0026 (2)	0.67401 (17)	0.0246 (6)	
H6A	0.0809	−0.0159	0.7331	0.029*	
H6B	0.0954	−0.0837	0.6394	0.029*	
C7	0.22848 (19)	0.0459 (2)	0.70248 (17)	0.0215 (6)	
H7A	0.2824	−0.0132	0.7490	0.026*	
H7B	0.2522	0.0504	0.6437	0.026*	
C8	0.23979 (19)	0.1747 (2)	0.74873 (15)	0.0176 (5)	
H8A	0.2232	0.1680	0.8107	0.021*	
H8B	0.3222	0.2043	0.7646	0.021*	
C9	0.32287 (19)	0.5977 (2)	0.56932 (16)	0.0198 (5)	
C10	0.2842 (2)	0.5668 (2)	0.46137 (16)	0.0271 (6)	
H10A	0.3038	0.4793	0.4529	0.041*	
H10B	0.3250	0.6223	0.4295	0.041*	
H10C	0.1987	0.5790	0.4320	0.041*	
C11	0.2945 (2)	0.7356 (2)	0.58157 (17)	0.0275 (6)	
H11A	0.2090	0.7489	0.5533	0.041*	
H11B	0.3349	0.7893	0.5481	0.041*	
H11C	0.3214	0.7569	0.6511	0.041*	
C12	0.45464 (18)	0.5736 (2)	0.61593 (17)	0.0283 (6)	
H12A	0.4815	0.6038	0.6835	0.042*	
H12B	0.4973	0.6182	0.5792	0.042*	
H12C	0.4701	0.4833	0.6152	0.042*	
H1N	0.1857 (18)	0.544 (2)	0.5961 (15)	0.024 (7)*	
N1	0.25541 (17)	0.51356 (18)	0.61717 (13)	0.0169 (5)	
N2	0.15788 (14)	0.26976 (16)	0.68447 (12)	0.0126 (4)	
Si	0.29025 (5)	0.49627 (6)	0.74618 (5)	0.01692 (15)	
Zn	0.20862 (2)	0.32732 (2)	0.565755 (18)	0.01447 (7)	

### Atomic displacement parameters (Å^2^)

**Table:**

	*U*^11^	*U*^22^	*U*^33^	*U*^12^	*U*^13^	*U*^23^
Br1	0.01765 (13)	0.02519 (15)	0.01490 (13)	−0.00001 (11)	0.00222 (10)	0.00006 (11)
Br2	0.02695 (14)	0.02831 (15)	0.03360 (15)	0.01126 (12)	0.01917 (12)	0.00731 (13)
C1	0.0218 (14)	0.0238 (15)	0.0254 (15)	−0.0039 (11)	0.0025 (12)	0.0022 (12)
C2	0.0291 (15)	0.0299 (16)	0.0306 (16)	−0.0056 (12)	0.0112 (13)	−0.0105 (12)
C3	0.0175 (13)	0.0198 (14)	0.0152 (13)	0.0025 (11)	0.0081 (11)	−0.0013 (11)
C4	0.0144 (12)	0.0245 (14)	0.0156 (13)	−0.0032 (11)	0.0062 (11)	0.0008 (11)
C5	0.0199 (14)	0.0276 (16)	0.0214 (14)	−0.0114 (12)	0.0052 (12)	−0.0033 (12)
C6	0.0311 (15)	0.0174 (14)	0.0265 (15)	−0.0076 (12)	0.0115 (13)	−0.0007 (11)
C7	0.0254 (14)	0.0150 (14)	0.0252 (14)	0.0025 (11)	0.0099 (12)	0.0047 (11)
C8	0.0152 (12)	0.0183 (14)	0.0175 (13)	0.0015 (11)	0.0030 (10)	0.0054 (11)
C9	0.0178 (13)	0.0194 (14)	0.0220 (14)	−0.0037 (11)	0.0065 (11)	0.0027 (11)
C10	0.0347 (16)	0.0228 (15)	0.0268 (15)	−0.0032 (12)	0.0144 (13)	0.0059 (12)
C11	0.0279 (15)	0.0201 (15)	0.0353 (16)	−0.0060 (12)	0.0118 (13)	−0.0006 (12)
C12	0.0176 (14)	0.0345 (17)	0.0342 (16)	−0.0016 (12)	0.0106 (13)	0.0053 (12)
N1	0.0135 (11)	0.0187 (12)	0.0190 (11)	−0.0038 (9)	0.0064 (10)	−0.0013 (9)
N2	0.0101 (10)	0.0145 (11)	0.0121 (10)	−0.0007 (8)	0.0023 (8)	−0.0006 (8)
Si	0.0168 (4)	0.0177 (4)	0.0158 (4)	−0.0018 (3)	0.0049 (3)	−0.0033 (3)
Zn	0.01415 (14)	0.01651 (15)	0.01335 (15)	0.00006 (12)	0.00541 (12)	−0.00062 (12)

### Geometric parameters (Å, °)

**Table:**

Br1—Zn	2.3887 (4)	C7—C8	1.513 (3)
Br2—Zn	2.3622 (3)	C7—H7A	0.9900
C1—Si	1.850 (2)	C7—H7B	0.9900
C1—H1A	0.9800	C8—N2	1.500 (2)
C1—H1B	0.9800	C8—H8A	0.9900
C1—H1C	0.9800	C8—H8B	0.9900
C2—Si	1.850 (2)	C9—C10	1.517 (3)
C2—H2A	0.9800	C9—C12	1.520 (3)
C2—H2B	0.9800	C9—N1	1.526 (3)
C2—H2C	0.9800	C9—C11	1.532 (3)
C3—N2	1.504 (3)	C10—H10A	0.9800
C3—Si	1.884 (2)	C10—H10B	0.9800
C3—H3A	0.9900	C10—H10C	0.9800
C3—H3B	0.9900	C11—H11A	0.9800
C4—N2	1.496 (2)	C11—H11B	0.9800
C4—C5	1.512 (3)	C11—H11C	0.9800
C4—H4A	0.9900	C12—H12A	0.9800
C4—H4B	0.9900	C12—H12B	0.9800
C5—C6	1.524 (3)	C12—H12C	0.9800
C5—H5A	0.9900	N1—Si	1.7909 (19)
C5—H5B	0.9900	N1—Zn	2.1276 (19)
C6—C7	1.520 (3)	N1—H1N	0.85 (2)
C6—H6A	0.9900	N2—Zn	2.1096 (16)
C6—H6B	0.9900		
			
Si—C1—H1A	109.5	C10—C9—C12	109.55 (19)
Si—C1—H1B	109.5	C10—C9—N1	108.78 (18)
H1A—C1—H1B	109.5	C12—C9—N1	109.40 (18)
Si—C1—H1C	109.5	C10—C9—C11	109.01 (19)
H1A—C1—H1C	109.5	C12—C9—C11	110.46 (19)
H1B—C1—H1C	109.5	N1—C9—C11	109.62 (17)
Si—C2—H2A	109.5	C9—C10—H10A	109.5
Si—C2—H2B	109.5	C9—C10—H10B	109.5
H2A—C2—H2B	109.5	H10A—C10—H10B	109.5
Si—C2—H2C	109.5	C9—C10—H10C	109.5
H2A—C2—H2C	109.5	H10A—C10—H10C	109.5
H2B—C2—H2C	109.5	H10B—C10—H10C	109.5
N2—C3—Si	114.88 (13)	C9—C11—H11A	109.5
N2—C3—H3A	108.5	C9—C11—H11B	109.5
Si—C3—H3A	108.5	H11A—C11—H11B	109.5
N2—C3—H3B	108.5	C9—C11—H11C	109.5
Si—C3—H3B	108.5	H11A—C11—H11C	109.5
H3A—C3—H3B	107.5	H11B—C11—H11C	109.5
N2—C4—C5	112.22 (17)	C9—C12—H12A	109.5
N2—C4—H4A	109.2	C9—C12—H12B	109.5
C5—C4—H4A	109.2	H12A—C12—H12B	109.5
N2—C4—H4B	109.2	C9—C12—H12C	109.5
C5—C4—H4B	109.2	H12A—C12—H12C	109.5
H4A—C4—H4B	107.9	H12B—C12—H12C	109.5
C4—C5—C6	111.29 (19)	C9—N1—Si	124.57 (15)
C4—C5—H5A	109.4	C9—N1—Zn	120.32 (13)
C6—C5—H5A	109.4	Si—N1—Zn	102.34 (9)
C4—C5—H5B	109.4	C9—N1—H1N	102.9 (15)
C6—C5—H5B	109.4	Si—N1—H1N	105.6 (14)
H5A—C5—H5B	108.0	Zn—N1—H1N	96.6 (16)
C7—C6—C5	108.47 (18)	C4—N2—C8	108.62 (16)
C7—C6—H6A	110.0	C4—N2—C3	107.58 (15)
C5—C6—H6A	110.0	C8—N2—C3	108.60 (16)
C7—C6—H6B	110.0	C4—N2—Zn	114.54 (13)
C5—C6—H6B	110.0	C8—N2—Zn	113.37 (12)
H6A—C6—H6B	108.4	C3—N2—Zn	103.75 (12)
C8—C7—C6	111.13 (18)	N1—Si—C1	112.86 (10)
C8—C7—H7A	109.4	N1—Si—C2	114.35 (10)
C6—C7—H7A	109.4	C1—Si—C2	110.18 (11)
C8—C7—H7B	109.4	N1—Si—C3	97.42 (9)
C6—C7—H7B	109.4	C1—Si—C3	113.57 (10)
H7A—C7—H7B	108.0	C2—Si—C3	107.88 (10)
N2—C8—C7	113.13 (18)	N2—Zn—N1	95.62 (7)
N2—C8—H8A	109.0	N2—Zn—Br2	115.47 (5)
C7—C8—H8A	109.0	N1—Zn—Br2	112.03 (5)
N2—C8—H8B	109.0	N2—Zn—Br1	107.97 (5)
C7—C8—H8B	109.0	N1—Zn—Br1	106.69 (5)
H8A—C8—H8B	107.8	Br2—Zn—Br1	116.759 (13)
